# Genome-wide identification and expression profiling analysis of *DIR* gene family in *Setaria italica*

**DOI:** 10.3389/fpls.2023.1243806

**Published:** 2023-09-20

**Authors:** Luping Gong, Bingbing Li, Tao Zhu, Baoping Xue

**Affiliations:** ^1^ College of Life Science and Engineering, Henan University of Urban Construction, Pingdingshan, China; ^2^ State Key Laboratory of Hybrid Rice, Department of Plant Sciences, College of Life Sciences, Wuhan University, Wuhan, China

**Keywords:** dirigent gene family, evolution, expression analysis, stress responses, *Setaria italica*

## Abstract

Dirigent (DIR) proteins play essential roles in regulating plant growth and development, as well as enhancing resistance to abiotic and biotic stresses. However, the whole-genome identification and expression profiling analysis of *DIR* gene family in millet *(Setaria italica (Si))* have not been systematically understood. In this study, we conducted genome-wide identification and expression analysis of the *S. italica DIR* gene family, including gene structures, conserved domains, evolutionary relationship, chromosomal locations, *cis*-elements, duplication events, gene collinearity and expression patterns. A total of 38 *SiDIR* members distributed on nine chromosomes were screened and identified. SiDIR family members in the same group showed higher sequence similarity. The phylogenetic tree divided the *SiDIR* proteins into six subfamilies: DIR-a, DIR-b/d, DIR-c, DIR-e, DIR-f, and DIR-g. According to the tertiary structure prediction, DIR proteins (like SiDIR7/8/9) themselves may form a trimer to exert function. The result of the syntenic analysis showed that tandem duplication may play the major driving force during the evolution of *SiDIRs*. RNA-seq data displayed higher expression of 16 *SiDIR* genes in root tissues, and this implied their potential functions during root development. The results of quantitative real-time PCR (RT-qPCR) assays revealed that SiDIR genes could respond to the stress of CaCl_2_, CdCl, NaCl, and PEG6000. This research shed light on the functions of SiDIRs in responding to abiotic stress and demonstrated their modulational potential during root development. In addition, the membrane localization of SiDIR7/19/22 was confirmed to be consistent with the forecast. The results above will provide a foundation for further and deeper investigation of DIRs.

## 1 Introduction

Gramineae family member of millet (*Setaria italica*) originated in China. The prolific yield and diverse ecological niches of millet are in sharp contrast to its small stature and short life cycle (Li and Brutnell, 2011). The remarkable drought tolerance and wide-ranging germplasm collection of millet have provided ideal model systems for the studies of C_4_ evolution, comparative grass genomics, and biofuel feedstocks (Doust et al., 2009; Brutnell et al., 2010).

Dirigent (DIR) proteins were first reported and isolated in *Forsythia intermedia* and were found to be a model for regioselective and stereoselective coupling during biological processes (Davin et al., 1997; Davin and Lewis, 2005). Since then, DIR proteins have been more and more cloned and studied in seed plants, including *Thuja plicata*, *Schisandra chinensis* (Kim et al., 2012), *Pisum sativum* (Seneviratne et al., 2015), *Linum usitatissimum* (Corbin et al., 2018), *Glycine max* (Li et al., 2017), *Arabidopsis* (Gasper et al., 2016; Yonekura-Sakakibara et al., 2021), rice (Duan et al., 2023), and cotton (Liu et al., 2021). According to the previous reports, DIR proteins in seed plants could be divided into six different subfamilies, named DIR-a and DIR-like groups (b/d, c, e, f, and g) (Ralph et al., 2007). The protein crystal structures of (+)-DIR (PsDRR206) and (−)-DIR (AtDIR6) have been obtained. The DIR protein is composed of eight reverse parallel β spiral structures connected in series β-barrel, which exists in the form of a trimer (Kim et al., 2015; Gasper et al., 2016).

The number of *DIR* and *DIR-like* genes is different in distinct plant species: 26 in *Arabidopsis* (Paniagua et al., 2017), 19 in *Isatis indigotica* (Li et al., 2014), and 55 in rice (Duan et al., 2023). Also, *DIR* genes are expressed variously in plant tissues including but not limited to leaves and roots (Paniagua et al., 2017). The promoter activity of *DIR* genes has been detected primarily in the vascular bundle of red cedar (Kim et al., 2002). Additionally, five conserved motifs (motifs I–V) have been identified within the DIR and DIR-like protein sequences in *Arabidopsis*, spruce, and rice (Ralph et al., 2007; Duan et al., 2023). It is worth mentioning that the DIR domain within ESB1 protein is indispensable for the lignin deposition of Casparian strips (Hosmani et al., 2013), and the *N*-glycosylation sites of Asn were reported to be representative amino acids of FiDIR1 (Burlat et al., 2001). Except for this, some DIR proteins were proposed to mediate the formation of gossypol in cotton (Effenberger et al., 2015; Effenberger et al., 2017; Lin et al., 2023). DIR proteins in leguminous plants showed dehydratase activity, although lacking a catalytic active center (Uchida et al., 2017).

The rapid progress rate of whole-genome sequencing projects also gives opportunities to resolve agricultural difficulties (Schnable et al., 2009; Li and Brutnell, 2011). Increasing findings suggest that DIRs also have various developmental regulation roles. For instance, the expression level of *ScDIR* genes was induced by PEG6000 and NaCl stresses (Guo et al., 2012). Most *VrDIR* genes varied their expression levels under high salt and drought stresses (Xu et al., 2021). The silencing of *CaDIR7* reduced peppers’ tolerance to *Phytophthora capsici* and abiotic stress (Khan et al., 2018). Recent research showed that *GhDIR5* mutation prevented gossypol formation in cotton (Guo et al., 2012). DIR members also function in regulating lignin biosynthesis to stand and defend against microorganisms and insects (Burlat et al., 2001; Hosmani et al., 2013). The resistance to *Phytophthora sojae* and the total lignan accumulation of *GmDIR22* overexpressor were significantly enhanced (Li et al., 2017). After being infected by *Fusarium solani*, the transcriptional level of *PsDRR266* was induced (Seneviratne et al., 2015). These similar benefits can be also found in *GhDIR* overexpression plants (Shi et al., 2012). Therefore, DIR proteins are necessary during the diverse biological and physiological processes of plants. The whole-genome identification of DIR protein is practicable for us to enable plant tolerance. However, an understanding of DIR proteins in *S. italica* is lacking.

In this study, genome-wide analysis of 38 *DIR* gene families in *S. italica* (*SiDIR*s) has been performed using bioinformatics methods. The evolutionary history was investigated through their phylogenetic relationships, sequence similarity, gene structure features, motif positions, tertiary structure, chromosome distributions, *cis*-elements, duplication events, and gene collinearity. The comprehensive expression patterns of *SiDIR*s in the five tissues of flag leaf, stem, root, panicle, and mesophyll unravel their important regulatory roles during millet development. Also, the active transcriptional responses of *SiDIR* genes to abiotic stress highlight the functional potential involved in these physiological processes. The finding of interacting proteins will provide candidate members that are involved in the adaptivity of millets to various environments. Additionally, the analysis of subcellular localization illustrates the potential function of SiDIRs in cell membranes. Our studies will provide useful theoretical support and new insights into exploring millet resistance and crop yield potential.

## 2 Results

### 2.1 Identification of DIR family members in *S. italica*

A total of 38 DIR and DIR-like members in *S. italica* were confirmed by HMMER and BLASTP methods. According to their chromosomal location, the 38 DIR genes were named SiDIR1 to SiDIR38. The protein sequence length of all the SiDIRs was less than 350 amino acids, ranging from 158 (SiDIR10) to 340 (SiDIR38); the molecular weight ranged from 16.8 (SiDIR8) to 34.9 (SiDIR7) kDa; the theoretical PI ranged from 4.79 (SiDIR38) to 9.82 (SiDIR9) (**Supplemental Data 1**). The predicted subcellular localization of most SiDIR proteins was in the cell membrane, while the remaining family members were located in the cell wall, chloroplast, mitochondrion, nucleus, etc. (**Supplemental Data 1**). The *N*-glycosylation sites of SiDIR protein sequences were also analyzed. It is interesting to find that 60% of proteins have the distribution of *N*-glycosylation sites (**Supplemental Data 1**). These results showed the divergence of millet *DIR* genes and hinted at their distinct functional potential.

### 2.2 Phylogenetic analysis and sequence similarity of SiDIRs

**Figure 1:**
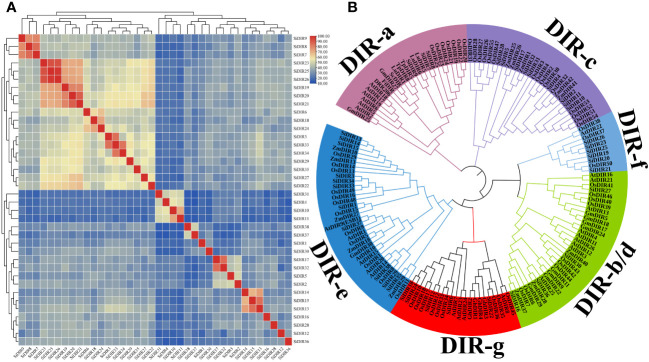
Sequence similarity analysis and the phylogenetic tree of SiDIRs. **(A)** The heatmap of SiDIR sequence similarity. The protein pairwise similarity matrix was obtained and visualized using TBtools software. The color indicates the similarity percentage, and the color scale values are shown on the upper right. **(B)** Phylogenetic tree of SiDIRs, AtDIRs, OsDIRs, ZmDIRs, GmDIRs, GhDIRs, LuDIRs, TpDIRs, FiDIR, and ScDIR. The evolutionary tree was constructed by neighbor-joining method (bootstrap values: 1,000 replicates). Pink purple, green, purple, blue, pale blue, and red represent the subgroup of DIR-a, DIR-b/d, DIR-c, DIR-e, DIR-f, and DIR-g, respectively.

To analyze the homology of SiDIRs, we used ClustalW and found that the protein sequences within the same subfamily have high similarity (**Figure 1A**). For example, SiDIR7, SiDIR8, and SiDIR9 have high sequence similarity (78%–86%); SiDIR19, SiDIR20, SiDIR21, SiDIR23, SiDIR25, and SiDIR26 have high sequence similarity (72%–89%). Also, we found that individual amino acids within the conserved motifs have undergone specific mutations. The amino acid of SiDIR35 changed from “FG” to “FS”, and SiDIR2, SiDIR10, and SiDIR11 changed from “FG” to “LG”. In addition, the conservative amino acid of SiDIR2, SiDIR5, SiDIR8, SiDIR9, SiDIR10, and SiDIR11 changed from “VGRAQG” to “VARAQG” (**Supplemental Data 2**). Due to the differences in amino acid sequences, different DIR proteins may have diverse functions.

To further elucidate the evolutionary relationship of SiDIRs, a phylogenetic tree containing 38 SiDIRs, 26 AtDIRs, 55 OsDIRs, 13 ZmDIRs, 11 GmDIRs, 4 GhDIRs, 3 LuDIRs, 2 TpDIRs, 1 FiDIR, and 1 ScDIR were constructed (**Figure 1B**). The DIR members could be grouped into DIR-a, DIR-b/d, DIR-c, DIR-e, DIR-f, and DIR-g. DIR-e (blue in **Figure 1B**) contained the largest number at 11 SiDIRs, while DIR-a (pink purple in **Figure 1B**) contained the smallest number at three SiDIRs. DIR-b/d (green in **Figure 1B**), DIR-c (purple in **Figure 1B**), DIR-f (pale blue in **Figure 1B**), and DIR-g (red in **Figure 1B**) contained four, nine, six, and five SiDIRs, respectively. Interestingly, no AtDIR member was found in the subgroup of DIR-c.

### 2.3 Analysis of gene structures, protein domains, and conserved motifs

**Figure 2:**
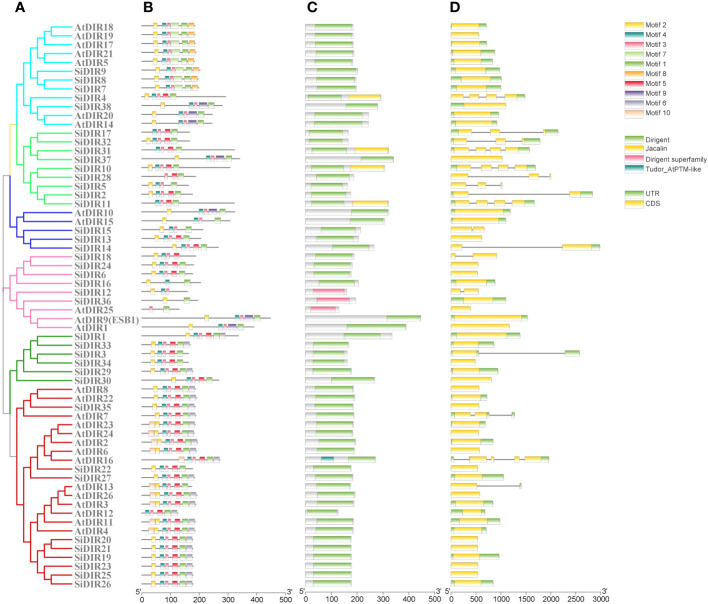
Analysis of gene structure, protein domains, and conserved motifs of SiDIRs. **(A)** Phylogenetic tree of SiDIRs and AtDIRs. **(B)** The conserved motifs of SiDIRs that were predicted by MEME. **(C)** The conserved domains were predicted and analyzed by NCBI-CDD. **(D)** Exo-intron distribution of *SiDIR* genes. This picture was visualized using TBtools software.

We used NCBI-CDD and MEME databases to analyze the gene structure and the distribution of conserved motifs, respectively. We found that the gene structure and conserved motifs of SiDIRs were similar within the same subgroups (**Figures 2A–D**). For example, SiDIR4, SiDIR7, SiDIR8, SiDIR9, and SiDIR38 contained five to six identical conserved motifs (**Figure 2B**). Except for the dirigent domains, the DIR family also contains three other specific domains including jacatin, dirigent superfamily domain, and tudor_AtPTM-like domain (**Figure 2C**). Some DIR-c subfamily members contain an N-terminal DIR domain and C-terminal end of a jacalin-related lectin (JRL) domain (**Figure 2C**), which show high specificity to bind mono- or oligo-saccharides (Kittur et al., 2007; Huwa et al., 2021; Huwa et al., 2022). Among the 38 SiDIRs, four members contained the jacatin domain, two members contained the dirigent superfamily domain, and no member contained the tudor_AtPTM-like domain (**Figure 2C**). Additionally, nine members (*SiDIR17*, *SiDIR32*, *SiDIR31*, *SiDIR37*, *SiDIR10*, *SiDIR28*, *SiDIR5*, *SiDIR2*, and *SiDIR11*) contained introns ranging from one to three, which is higher than that of the other *DIR* members. Most of the *DIR* and *DIR-like* family members contained exons ranging from two to three (**Figure 2D**). The differences in gene structure or conserved motifs might be due to various biological functions of SiDIRs.

### 2.4 Amino acid alignment and the tertiary structure prediction of SiDIR7/8/9

**Figure 3:**
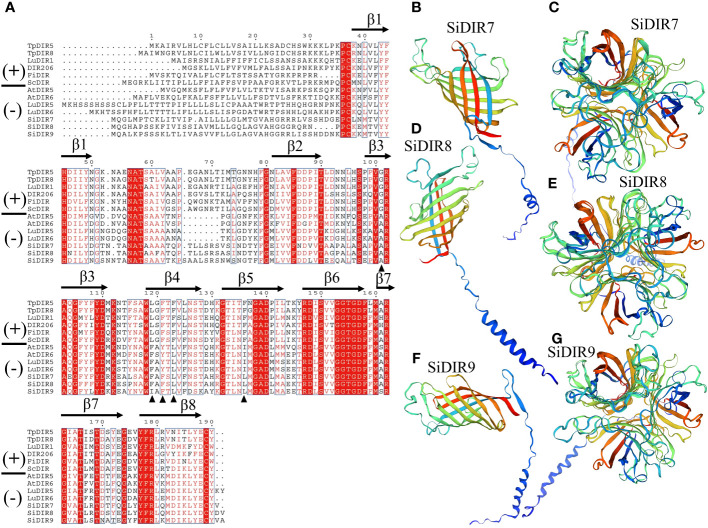
Amino acid alignment and the tertiary structure prediction of SiDIR7, SiDIR8, and SiDIR9. **(A)** The sequence alignment of SiDIR7, SiDIR8, and SiDIR9 with (−)- and (+)-DIRs. The black triangle indicates residues that are differently conserved in (+)- and (−)-DIRs. **(B–G)** Predicted tertiary structures of SiDIR proteins.

FiDIR1 and DRR206 participate in the formation of (+)-pinoresinol (Pickel et al., 2010; Seneviratne et al., 2015). AtDIR6 and LuDIR5/LuDIR6 are able to form (−)-pinoresinol (Dalisay et al., 2015; Gasper et al., 2016). Based on the results, SiDIR7, SiDIR8, SiDIR9, TpDIR5/8, LuDIR1/5/6, AtDIR5/6, FiDIR, and ScDIR belonged to the same DIR-a subfamily (**Figure 1**). We aligned the amino acids and found that some sites were highly conserved, such as alanine (A) located in β3 (**Figure 3A**). Phenylalanine (F) sites located in the β4 structure were similar, except for their mutation to isoleucine (I) in SiDIR9 (**Figure 3A**). Also, the hydrophilic amino acid “Y” located in the β4 structure mutated into unhydrophobic amino acid “F” (phenylalanine), while the hydrophobic amino acid “I” located in β5 structure mutated to hydrophobic amino acid “L” (leucine) (**Figure 3A**).

It has been known that the tertiary structure of the AtDIR6 trimer is an eight-stranded antiparallel β-barrel with spatially well-separated cavities for substrate binding. The binding cavity is composed of two lobes, and each of the two lobes is lined with a set of hydrophilic and potentially catalytic residues that are conserved in (+)- and (−)-pinoresinol-forming DIRs (Gasper et al., 2016). A further forecast showed that the tertiary structure of SiDIR7, SiDIR8, and SiDIR9 consisted of eight-stranded antiparallel β-barrels (**Figures 3B, D, F**). SiDIR7/8/9 were able to form trimers by themselves through homologous modeling prediction (**Figures 3C, E, G**). These suggested that SiDIR7, SiDIR8, and SiDIR9 may be able to direct the formation of (−)-pinoresinol. Meanwhile, the different locations of hydrophobic cavities and diverse substrate binding sites (**Figures 3B–G**) also potentially revealed their different functions.

### 2.5 Duplication event analysis of *SiDIR* genes

**Figure 4:**
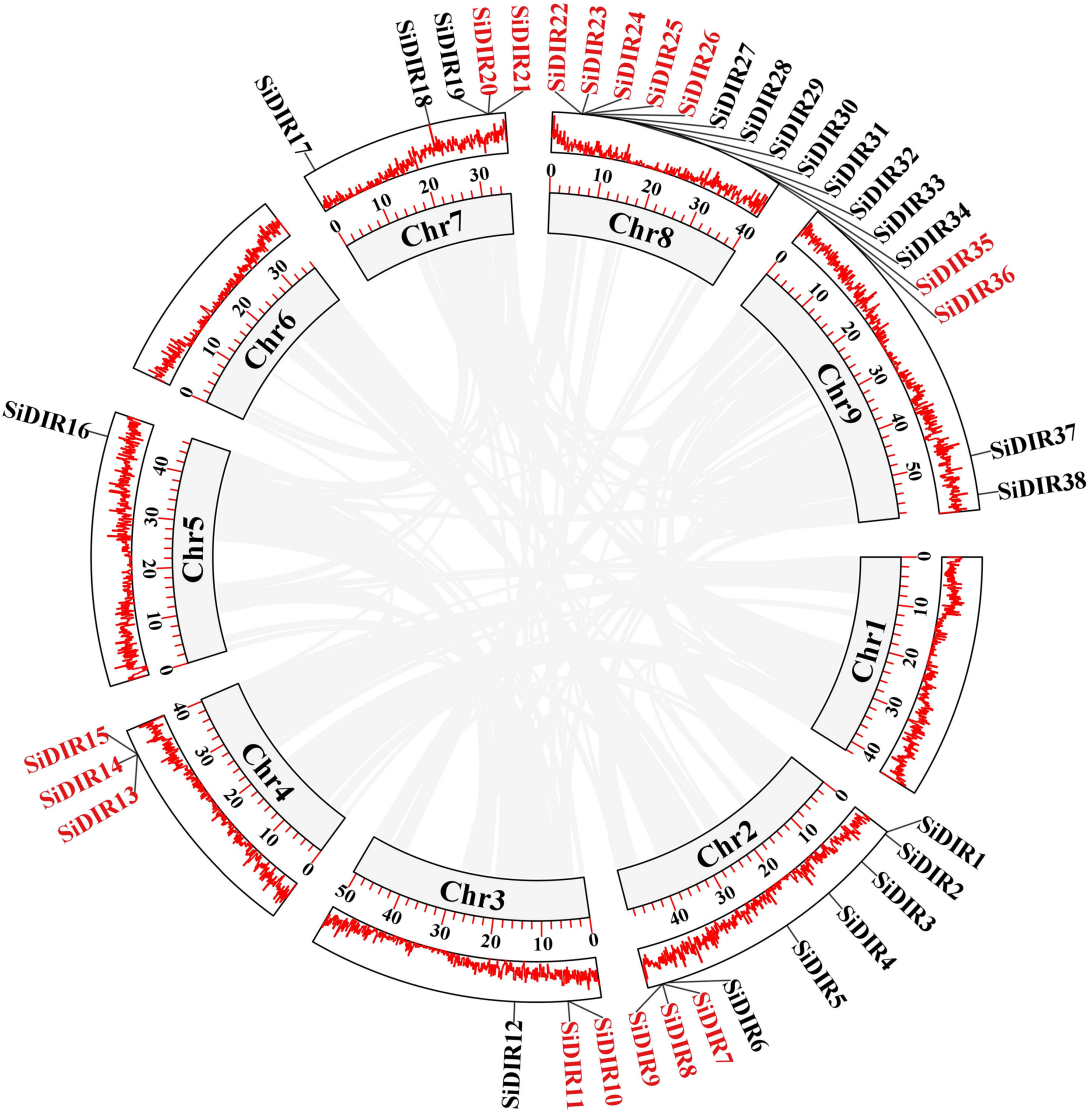
The gene location and duplication events of *SiDIR* genes. The tandem duplicated genes are indicated in red color.

According to genome annotation, we analyzed the distribution of 38 *SiDIR* genes. The 38 *SiDIR* genes were randomly distributed on nine chromosomes (Chr) (**Figure 4**). Both Chr3 and Chr4 contain three members (~7.89%), while Chr9 contains two members (~5.26%). Compared to Chr5 (1 gene, ~2.63%), Chr8 contained the largest number of *SiDIR* family (15 genes, ~39.47%), which appears in the form of gene clusters (**Figure 4**). Although there was no distribution on the first and sixth chromosomes, most *SiDIR* genes were distributed on the ends of the chromosomes.

Tandem duplication (TD) and whole-genome duplication (WGD)/segmental duplication (SD) of genes drive the evolution and expansion of gene family (Weidenbach et al., 2016; Zhang et al., 2022; Guo et al., 2023). We analyzed the duplication events of *SiDIR* genes and found 10 tandem duplication events involving 17 *SiDIR* genes on Chr2, 3, 4, 7, and 8 (**Figure 4**; **Supplemental Data 3**). The genes of *SiDIR7*/*8*/*9*, *SiDIR10*/*11*, *SiDIR13*/*15*, *SiDIR20*/*21*, and *SiDIR25*/*26* in tandem duplication events were from the same subgroup (**Figure 4**), indicating the accuracy of the group division of the phylogenetic tree. However, no genome duplication (WGD)/SD events were found. These results revealed that TD events contribute largely to expanding *SiDIR* gene family.

Meanwhile, we counted the Ka, Ks, and Ka/Ks ratios of the duplication gene pairs using DNASP to analyze the evolutionary selection of duplication pairs in *SiDIR* gene family. We found that the Ka/Ks ratios of most gene pairs were less than 1 (**Supplemental Data 3**), implying that these *SiDIR*s have undergone negative selection. Only two gene pairs, *SiDIR13* and *SiDIR14*, and *SiDIR23* and *SiDIR24* (**Supplemental Data 3**) had Ka/Ks ratios greater than 1, indicating that *SiDIR13* and *SiDIR14*, and *SiDIR23* and *SiDIR24* may undergo positive selection and that they are important for the evolution of millets.

### 2.6 Collinearity analysis of *SiDIR*s

**Figure 5:**
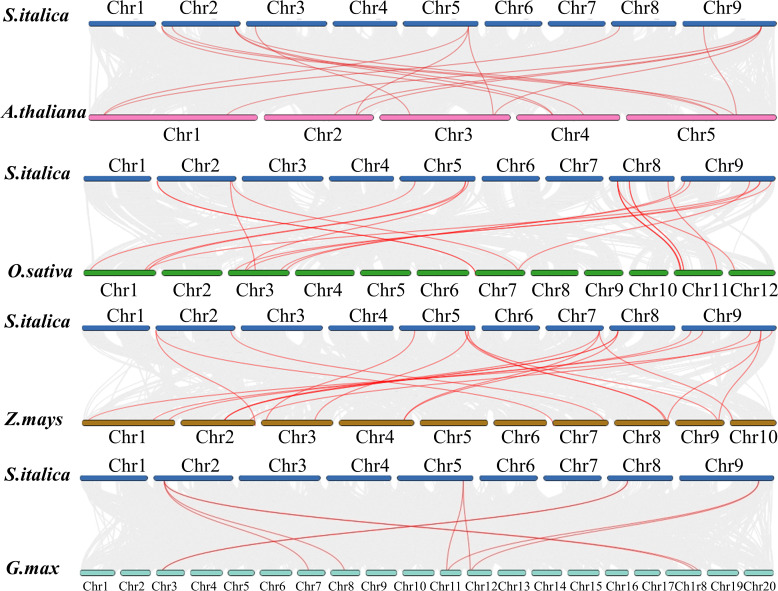
Syntenic analysis between *SiDIRs* and the *DIR* genes of *Arabidopsis thaliana*, *Oryza sativa*, *Zea mays*, and *Glycine max*. The collinear blocks are shown by gray lines, while the syntenic DIR homologous gene pairs are highlighted by red lines. “Chr1–20” means the chromosome number.

To investigate more deeply the evolution mechanisms of *SiDIR* genes, 12, 16, 14, and 8 ortholog *DIR* gene pairs were identified when compared millets with *Arabidopsis thaliana*, *Oryza sativa*, *Zea mays*, and *G. max*, respectively (**Figure 5**; **Supplemental Data 4**). Interestingly, we found that some *SiDIR* genes were identified to be associated with at least three homologous gene pairs, such as *SiDIR6 *to *AT4G11180.1, AT4G23690.1 *and* AT5G42500.1; SiDIR37 *to* AT1G65870.1, AT2G28670.1, AT2G39430.1 *and* AT3G55230.1; SiDIR3 *to* Glyma.07G157100.1, Glyma.08G258300.1, Glyma.18G282600.1 *and* Glyma.18G207700.1* (**Figure 5**; **Supplemental Data 4**). Strikingly, some collinear gene pairs identified between Si and Os, and Zm were not found between *Si* and *Os*, and *Zm* were not found between *Si* and *At*, and *Gm*, such as *SiDIR7/LOC_Os07g44250.1, *and* SiDIR7/Zm00001d022270* (**Figure 5**; **Supplemental Data 4**). Some collinear gene pairs identified between *Si* and *At* were not found between *Si* and *Os*, *Gm* and *Zm*, such as *SiDIR10/AT3G16450.1*. We speculated that these orthologous genes may exert vital roles during the evolutionary process of DIRs.

### 2.7 *cis*-Element analysis of *SiDIRs*

**Figure 6:**
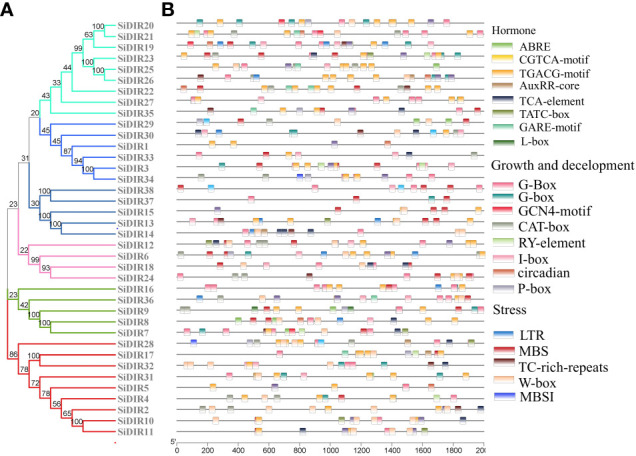
*cis*-Element analysis of *SiDIRs*. **(A)** Phylogenetic tree of SiDIRs. **(B)** *cis*-Element analysis of *SiDIR* genes; the colorful boxes represent different *cis*-elements.

To predict the functions and regulatory mechanisms of *SiDIR* genes, the *cis*-elements within their promoters were analyzed. A total of 23 different *cis*-elements in the promoter of *SiDIRs* were detected. These include *cis*-elements involved in phytohormone response (ABRE: abscisic acid, AuxRR-core: auxin, GARE-motif: gibberellin, CGTCA-motif: methyl jasmonate, and TCA-element: salicylic acid), light response, developmental regulation (circadian control, endosperm, flavonoid biosynthesis, and meristem), and environmental stress (defense and stress, drought, and low temperature) (**Figure 6**). In plants, these *cis*-elements regulate various signaling pathways, hinting at the complicated regulatory function of SiDIRs.

### 2.8 Interaction network of SiDIR proteins

In order to further clarify the functions and regulatory pathways among DIR family, a protein–protein interaction network of AtDIR family was analyzed and predicted by STRING software (**Figure S1**). Nearly all of the AtDIR proteins could interact with other members. AtDIR9 (ESB1), At4g13580, and At2g39430 were central to the interaction network. At4g13580 might interact with AtDIR9 (ESB1), At2g39430, At5g42500, At3g24020, At4g11190, and AtDIR6. Similarly, At4g11190 may also interact with At3g24020 and AtDIR6. The interaction protein numbers of At3g13650, At1g22900, and At5g42510 might be the least. Interestingly, the interaction between At5g42510 and At2g39430 might be strong (**Figure S1**). According to the protein similarity, we inferred that SiDIR family members might have a similar protein–protein interaction network to that of AtDIRs.

### 2.9 The expression analysis of *SiDIRs* in five different tissues

**Figure 7:**
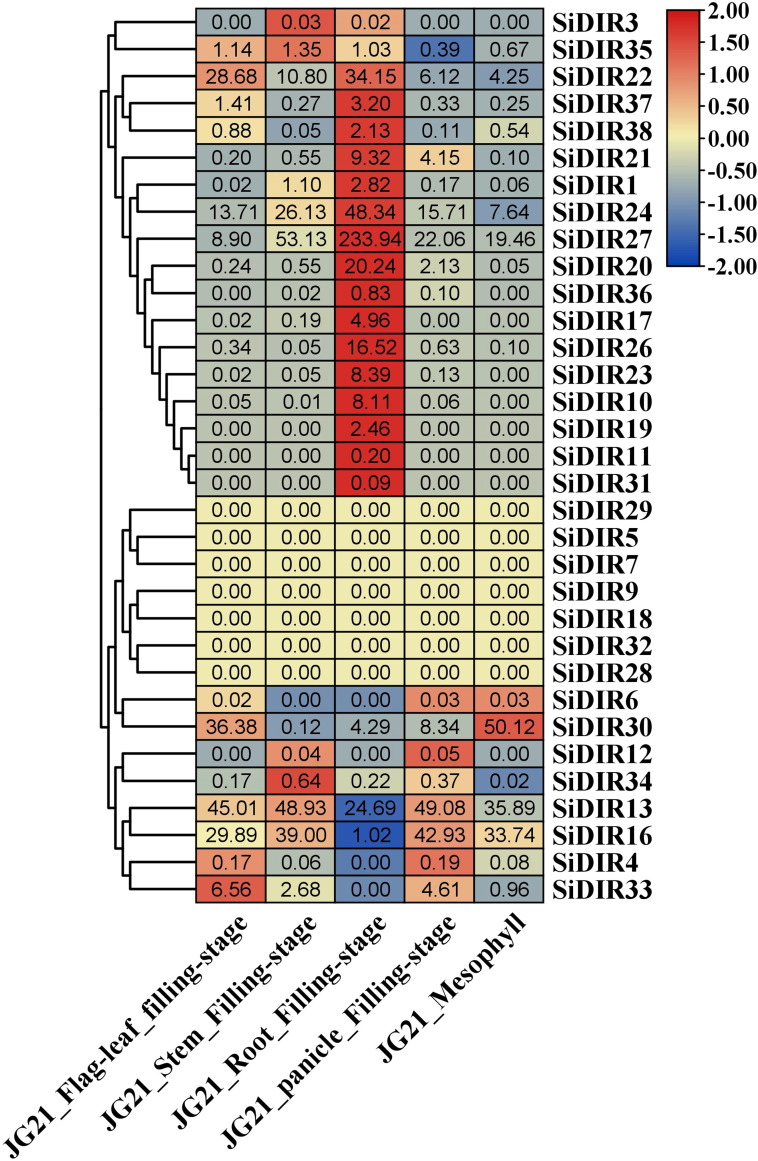
The heatmap of the expression profiles of *SiDIRs* in five tissues (Flag leaf, Stem, Root, Panicle, and Mesophyll). TPM values of *SiDIRs* were transformed by log2, and the heatmap was created by the software of TBtools.

To gain insights into the functions of SiDIRs, the expression patterns of SiDIRs were analyzed based on RNA‐seq data. A total of 16 SiDIR genes including *SiDIR1, SiDIR10, SiDIR11, SiDIR17, SiDIR19, SiDIR20, SiDIR21, SiDIR22, SiDIR23, SiDIR24, SiDIR26, SiDIR27, SiDIR31, SiDIR36, SiDIR37, *and* SiDIR38* expressed higher in root than other tissues (**Figure 7**; **Supplemental Data 5**). Unlike the expression of *SiDIR12*, which was detected mainly in the stem and panicle, that of *SiDIR3* was expressed mainly in the stem and root (**Figure 7**; **Supplemental Data 5**). *SiDIR10, SiDIR11, SiDIR19*, and *SiDIR31* are expressed only in root. Additionally, there were no transcripts of *SiDIR5, SiDIR7, SiDIR9, SiDIR18, SiDIR28, SiDIR29*, and *SiDIR32* being detected in the organs of flag leaf, stem, root, panicle, and mesophyll (**Figure 7**; **Supplemental Data 5**). These results hinted at the essential regulatory function of *SiDIRs* in plant development.

### 2.10 Expression analysis of *SiDIRs* under CaCl_2_, NaCl, CdCl, and PEG6000 treatments

**Figure 8:**
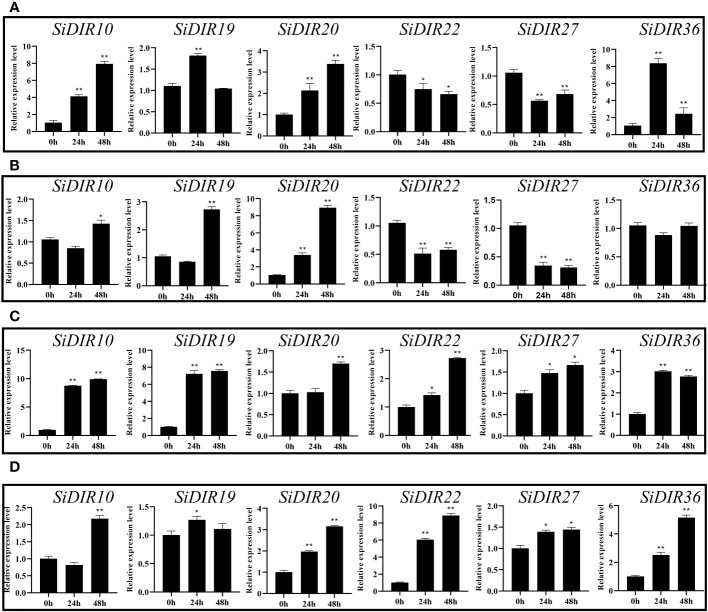
Expression patterns of six *SiDIR* genes under abiotic stresses. **(A)** 20 mM CaCl_2_ treatment, **(B)** 1 mM CdCl treatment, **(C)** 150 mM NaCl treatment, and **(D)** 10% PEG6000 treatment. The expression levels were calculated and shown as means ± SDs (n = 3). Statistically significant differences were analyzed by Student’s t-test (* p < 0.05, ** p < 0.01).

To further characterize the *SiDIR* genes in response to abiotic stresses, the millets were treated with CaCl_2_, NaCl, CdCl, and PEG6000, and the transcriptional analysis of six *SiDIRs* in roots was carried out. After treatment with 20 mM of CaCl_2_, the transcriptional level of *SiDIR19 *and* SiDIR36* reached the maximum at 24 h, while *SiDIR10 *and* SiDIR20* reached the maximum at 48 h (**Figure 8A**). Specifically, the expression level of *SiDIR10* (at 48 h) and *SiDIR36* (at 24 h) was approximately eight times higher than that of control. Unlike this, the relative expression of *SiDIR22 *and* SiDIR27* was downregulated (**Figure 8A**). For 1 mM CdCl treatment, the transcription level of *SiDIR10/19/20* was upregulated, while *SiDIR22/27* was significantly downregulated. However, the expression induction of *SiDIR36* was not obvious under CdCl treatment (**Figure 8B**). Upon treatment with 150 mM of NaCl, the expression of *SiDIR10/19/22/27/36* was upregulated at 24 h and 48 h, and *SiDIR20* was upregulated only at the time point of 48 h (**Figure 8C**). In addition, the expression levels of *SiDIR19/20/22/27/36* were upregulated after being treated with 10% PEG6000 for 24 h, and *SiDIR10* was not upregulated until being treated for 48 h (**Figure 8D**). These results showed that different *SiDIR* genes with different expression patterns may function differently during millet growth.

### 2.11 Gene co-expression analysis

**Figure 9:**
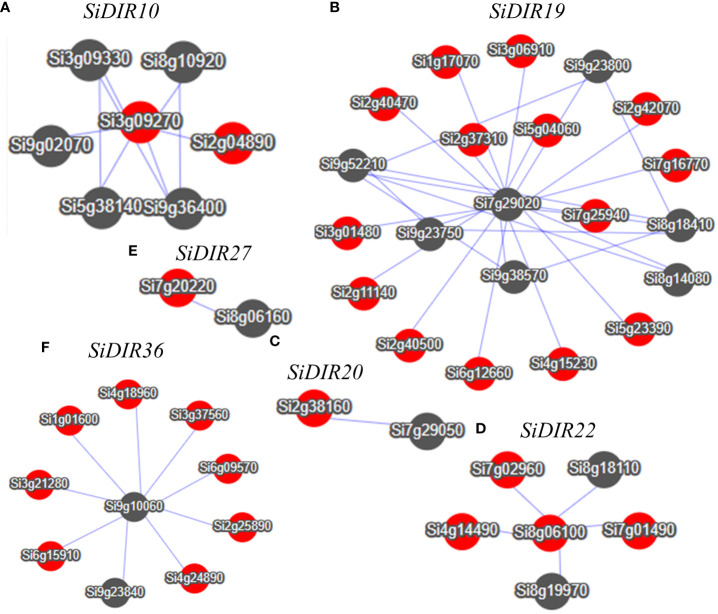
Co-expression network of *SiDIR10* **(A)**, *SiDIR19* **(B)**, *SiDIR20* **(C)**, *SiDIR22* **(D)**, *SiDIR27* **(E)**, and *SiDIR36* **(F)**. Dots represent genes, and lines indicate that they have co-expression relationship.

Co-expression analysis can help find genes that were closely co-regulated during the physiological process. Based on the *MDSI* database (Li et al., 2023), we constructed co-expression networks centered on the *SiDIR10, SiDIR19, SiDIR20, SiDIR22, SiDIR27, *and* SiDIR36*. As shown in **Figure 9**, we obtained a total of six co-expression networks. Among them, the network centered on *SiDIR19* is the largest (including 21 genes). In contrast, the network centered on *SiDIR20 *and* SiDIR27* is the smallest (including one gene).

As shown in **Supplemental Data 6**, the co-expressed gene network centered on *SiDIR10* is significantly enriched in the carbohydrate metabolic process, oxidation-reduction process, ion binding, response to cadmium ion, and oxidation-reduction process. The network centered around *SiDIR19* showed significant enrichment in disease resistance, sterol biosynthetic process, malate transport, metal ion binding, response to oxidative stress, cell wall biogenesis, cell wall organization process, response to salt stress, response to abscisic acid, phosphate starvation, and response to stimulus process. Meanwhile, the network centered on *SiDIR36* showed significant enrichment in response to water deprivation, ion binding, lignin catabolic process, cell wall biogenesis, and carbohydrate metabolic process. Overall, these findings present an interesting phenomenon that warrants further investigations.

### 2.12 Subcellular localization of SiDIR7/19/22

**Figure 10:**
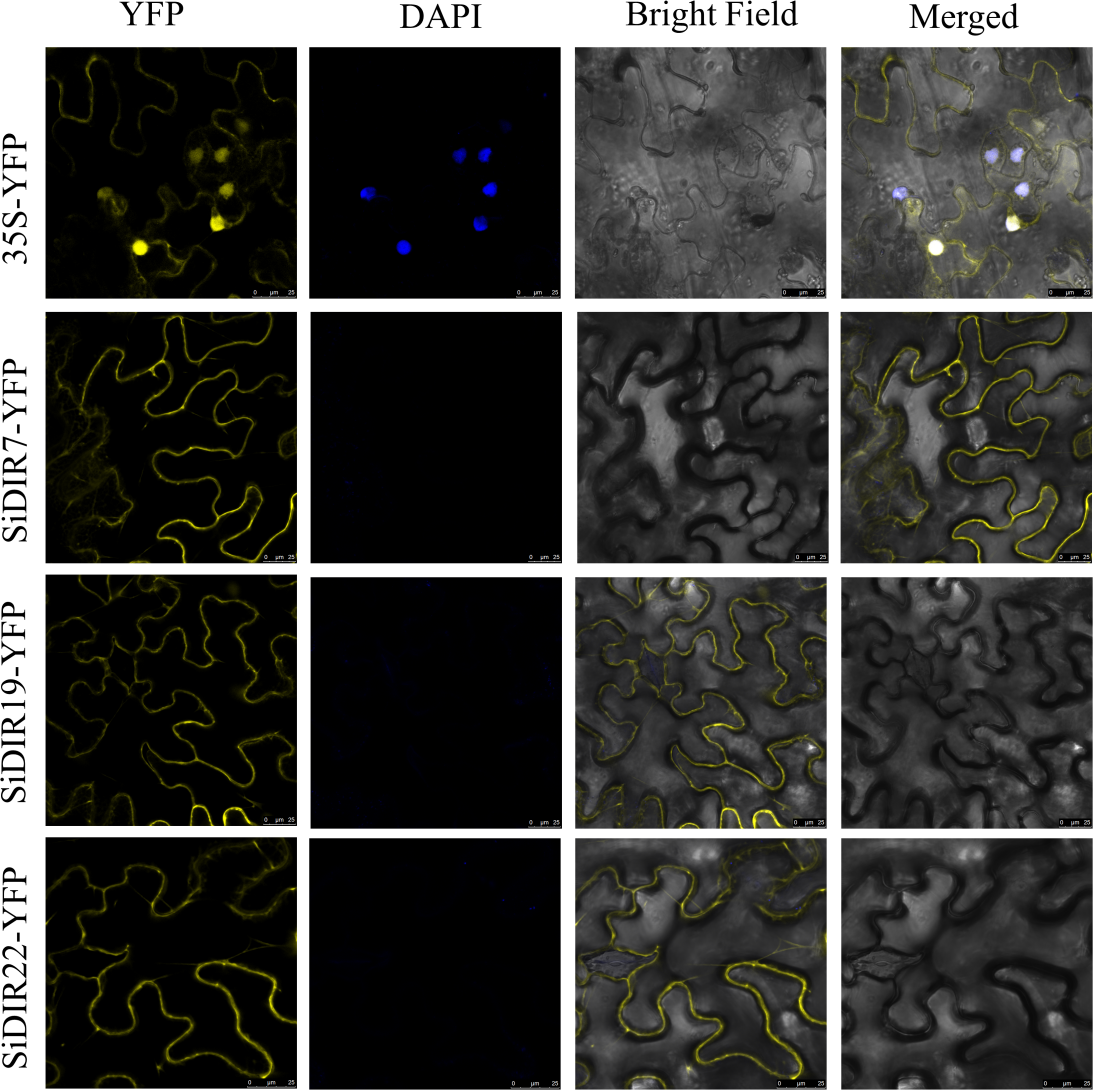
Subcellular localization analysis of SiDIR7/19/22 in Nicotiana tabacum L. leaves. Membrane localization of SiDIR7/19/22 was observed and confirmed by ×20 laser confocal microscopy, and 35S-YFP was the negative control. The blue fluorescence signal of DAPI indicated the nucleus. Scale bars: 25 µm.

To deeply analyze the function of SiDIRs, the fusion expression vectors of 35S-SiDIR7-YFP, 35S-SiDIR19-YFP, and 35S-SiDIR22-YFP were constructed, with an empty vector of 35S-YFP used as a negative control and DAPI fluorescence signal used to indicate the nucleus. These vectors were transferred into the leaves of Nicotiana tabacum L., and the fluorescence was observed by ×20 laser confocal microscopy. The negative YFP signal was expressed in the membrane and nucleus, while the fluorescence signals of 35S-SiDIR7-YFP, 35S-SiDIR19-YFP, and 35S-SiDIR22-YFP were mainly expressed in the cell membrane (**Figure 10**). This was coherent with the predicted analysis (**Supplemental Data 1**).

## 3 Discussion

Dirigent proteins are ubiquitous in all vascular plants, such as ferns, gymnosperms, and angiosperms (Davin and Lewis, 2000; Ralph et al., 2007; Li et al., 2014). Although *DIR* genes have been identified in many species including rice, cotton, *I. indigotica*, and pepper (Li et al., 2014; Paniagua et al., 2017; Khan et al., 2018; Liu et al., 2021; Duan et al., 2023), *SiDIR* gene family has not been comprehensively analyzed. In this study, we completely identified 38 DIR proteins that belonged to six groups in *S. italica*, investigated their evolutionary events of TD duplication, and explored their functional potential during root development and expression diversity when responding to various abiotic stresses.

Phylogenetic analysis suggested that DIR proteins of millet, *Arabidopsis*, rice, soybean, maize, cotton, etc., were divided into DIR-a, DIR-b/d, DIR-c, DIR-e, DIR-f, and DIR-g subgroups (**Figure 1**), which was consistent with the previous studies (Ralph et al., 2007; Khan et al., 2018; Duan et al., 2023). Specifically, the subgroup DIR-e contained the most DIRs (**Figure 1**). Members in the same subgroups have higher sequence similarity (**Figure 1**). Our conserved domain research showed that the dirigent superfamily domain only appeared in AtDIR25, SiDIR12, and SiDIR36 (**Figure 2C**). The gene structure analysis displayed the range of the introns from one to four, while the exons were from one to five (**Figure 2D**). This may be revealed that *DIR* genes gain or lose exons or introns during the process of chromosomal rearrangements.

A further amino acid alignment analysis (**Figure 3A**) displayed and confirmed the classification of SiDIRs (**Figure 1**). It has been shown that DIR-a subfamily members contain the well-characterized 8–8′-linked-lignan forming dirigent proteins. For example, AtDIR6 and LuDIR5/6 participate in the formation of (−)-pinoresinol (Dalisay et al., 2015; Gasper et al., 2016). SiDIR7, SiDIR8, and SiDIR9 were closely related to LuDIR5/6 (**Figure 1B**). SiDIR7, SiDIR8, and SiDIR9 contained the necessary conserved residues of alanine (A), phenylalanine (F), and leucine (L) that could form (−)-pinoresinol (**Figure 3A**). This analysis indicates that SiDIR7, SiDIR8, and SiDIR9 might be involved in the formation of (−)-pinoresinol. However, whether SiDIR7/8/9 influences crop resistance to biotic stress, like GmDIR22 or GhDIR1 by promoting lignan accumulation, deserves further investigations (Shi et al., 2012; Li et al., 2017). Except for this, the predicted tertiary structure of SiDIR7/8/9 (**Figures 3B–G**) was similar to that of AtDIR6 (Gasper et al., 2016), which illustrated the possibility of SiDIR7, SiDIR8, and SiDIR9 to form trimers, respectively. In addition, homologous proteins might have similar functions in different species. AtESB1 was necessary for the formation of the Casparian strip in roots (Hosmani et al., 2013). SiDIR37 was closely classified into the same subgroup as AtESB1 (**Figure 1B**), suggesting that SiDIR37 is potentially involved in Casparian strip formation. Also, impaired lignin deposition resulted in a defective CS barrier in the mutants of ZmESBL, thus increasing Na^+^ transport and salt sensitivity (Wang et al., 2022). Our future work will be focused on exploring the function of SiDIRs when moderating salt resistance.

The number of *SiDIR* genes (**Supplemental Data 1**) was more than that in *Arabidopsis* (Paniagua et al., 2017), pepper (Khan et al., 2018), and *I. indigotica* (Li et al., 2014). Conversely, it was less than that in rice, *Gossypium barbadense*, and *Gossypium hirsutum*, in which TD and SD/WGD may be the main driving force behind *DIR* gene family expansion (Liu et al., 2021; Duan et al., 2023). According to our analysis of gene duplication events and collinearity analysis, a total of 17 TD *SiDIR* genes were identified, although no SD/WGD was found (**Figures 4**, **5**). This revealed that tandem duplication may play an important role in expanding *SiDIR* gene family. Also, the number of tandem duplication genes (**Figure 4**) is considerable in the subgroups of DIR-f (**Figure 1B**), while the tandem duplication of the DIR-b/d group contributed to the expansion in pepper, cotton, spruce, and flax (Corbin et al., 2018; Khan et al., 2018; Liu et al., 2021; Duan et al., 2023). Based on the evolutionary functions of tandem duplication (Hanada et al., 2008), it is reasonable to infer that the rapid expansion of the DIR-f subfamily may be the adaptive evolution of millet. Interestingly, *SiDIR* genes were distributed unevenly on the chromosomes (except for chromosomes 1 and 6), and only *SiDIR16* was located on chromosome 5 (**Figure 4**). Because of the *DIR* duplication gene pairs of cotton and other plants (Liu et al., 2021), it is not surprising to find that the Ka/Ks ratios of most *SiDIR* duplication gene pairs were under 1 (**Supplemental Data 3**, **4**), which illustrated that the duplication gene pairs in millet were under purifying selection.

The diverse *cis*-elements of *SiDIR*s (**Figure 6**) could partially explain the diversified function of DIRs during plant development and plant defense against biotic and abiotic stresses (Baxter et al., 2009; Hosmani et al., 2013; Yang et al., 2021; Yonekura-Sakakibara et al., 2021; Wang et al., 2022).

It has been reported that 60% of the *AtDIR* genes show higher expression levels in roots compared with other organs (Paniagua et al., 2017). In millet, we found that approximately half of *SiDIR* genes similarly displayed higher expression levels in the root tissues (**Figure 7**). The expression pattern of *SiDIR*s is similar to that of most of the *OsDIR*s (Duan et al., 2023). In contrast, only a part of *VrDIR* genes was highly expressed in roots (Xu et al., 2021), implying the functional conservation and divergence of some *DIR* genes in different species. Considering universal *DIR* genes vary their number greatly in vascular plants, we supposed that DIRs are possibly the key family for aquatic plants to land.

Plants deal with abiotic stress to adapt to the circumstances and keep growing. Previous studies have confirmed that the expression of *DIR*s could respond to salt stress (Paniagua et al., 2017; Khan et al., 2018; Wang et al., 2022). For example, AtESB1 is involved in regulating the concentration of Na, S, K, As, Fe, Ca, Mn, and Zn in shoots (Baxter et al., 2009); *VrDIR*s are required in salt stress adjustment (Xu et al., 2021); the expression of *ScDIR* genes was induced by NaCl and PEG treatments (Guo et al., 2012); the transcription levels of *CaDIR4/7/12* were significantly regulated by NaCl or mannitol treatment (Khan et al., 2018). We found that the expression levels of *SiDIR10*/*19*/*20*/*22*/*27*/*36* could be induced by NaCl treatment (**Figure 8**). This hinted at the possibility of *SiDIR10*/*19*/*20*/*22*/*27*/*36* to be potential candidate genes coping with salt stress.

Lignin, deposited mostly in the secondary cell walls of vascular plants, contributes to water transport and plant stress responses (Hu et al., 2019; Oliveira et al., 2020; Dabravolski and Isayenkov, 2023). Combining the results in **Supplemental Data 6**, the co-expression network of *SiDIR36* illustrates its possible involvement in responding to salt or osmotic stresses by regulating lignin deposited in the cell walls. Unlike other family members, the expression levels of *SiDIR22*/*27* were downregulated when treated with CaCl_2_ and CdCl (**Figure 8**). We speculated that *SiDIR22* and *SiDIR27* may play synergistic regulation roles. Additionally, the co-expression network centered around *SiDIR27* exhibited significant enrichment in response to cytokinin and auxin-activated signaling pathways (**Figure 9**, **Supplemental Data 6**), indicating that *SiDIR27* may play a role in responding to salt stresses through plant hormone signal transduction. These results clarify the distinct and diverse function of DIRs under abiotic stresses. Meanwhile, the membrane localization of SiDIRs (**Figure 10**) was consistent with the prediction (**Supplemental Data 1**) and further revealed their potential vital roles during plant growth and development.

## 4 Materials and methods

### 4.1 Plant materials and treatments

Yugu 1 was used as the experimental material in this study. Millets were grown in a greenhouse in Wuhan, Hubei Province, China. Millets were grown in Hoagland nutrient solution (Li et al., 2022). For CaCl_2_, NaCl, CdCl, and PEG6000 treatments, millet seedlings were grown in Hoagland solution for 10 days and then treated with 20 mM of CaCl_2_, 150 mM of NaCl, 1 mM of CdCl, and 10% PEG6000, respectively. Plant roots were collected after treatment for 0 h, 24 h, and 48 h. Three biological replicates were carried out for each treatment.

### 4.2 Data sources and identification of DIRs in different species

The genome data of *A. thaliana*, *S. italica*, *Z. mays*, *G. max*, and *O. sativa* spp. *japonica* were downloaded from Ensembl Plants (http://plants.ensembl.org/index.html/). The AtDIR protein sequence was downloaded from TAIR (https://www.Arabidopsis.org/). The hidden Markov Model (HMM) file of the dirigent domain (PF03018) was downloaded as reported previously (Duan et al., 2023). HMMER 3.0 (E-value ≤ 1e^−5^, similarity > 50%) was used to search the DIR protein from the *S. italica* protein database. Further, based on the BLASTP method, we searched SiDIR protein sequences using AtDIR protein sequences (E-value ≤ 1e^−5^, similarity > 50%). All candidate DIR protein sequences were used to verify the DIR domain as analyzed previously (Duan et al., 2023). The longest transcript was obtained using the R package seqfinder (https://github.com/yueliu1115/seqfinder).

### 4.3 Phylogenetic analysis of SiDIRs and AtDIRs

The phylogeny tree of identified SiDIRs and DIRs of rice, *Arabidopsis*, maize, soybean, cotton, etc., were constructed using the neighbor-joining (NJ) method of MEGA7.0 (bootstrap: 1,000 replications) (Kumar et al., 2016). The website of iTOL (Interactive Tree of Life, https://itol.embl.de/) was used to enhance the evolutionary tree.

### 4.4 Sequence alignment and the tertiary structure prediction of SiDIR7/8/9

The sequence alignment of DIR proteins was carried out by ClustalW, and ESPript 3.0 (https://espript.ibcp.fr/ESPript/ESPript/) was used to illustrate the conserved residues. For the prediction of tertiary structure, the protein sequences were input and analyzed through homologous modeling in SWISS-MODEL (https://swissmodel.expasy.org/).

### 4.5 Gene structure and conserved motif analysis

The conserved motifs of *S. italica* DIR proteins were determined by MEME (http://meme-suite.org/) with a conserved motif number of 10. The gene structure information was acquired from GFF data. The conserved domains were obtained from NCBI-CDD (https://www.ncbi.nlm.nih.gov/Structure/cdd/wrpsb.cgi) and were subsequently visualized using TBtools software (Chen et al., 2020).

### 4.6 Gene duplication events and the analysis of Ka/Ks ratios

Segmental and tandem duplications were detected by MCScanX (Wang et al., 2012). The non-synonymous (Ka)/synonymous (Ks) ratios of duplication gene pairs were calculated using TBtools software. TBtools software was used to visualize the duplication events (Chen et al., 2020). The divergence time of all duplicate gene pairs was estimated as previously (Deng et al., 2019).

### 4.7 Expression pattern analysis of *SiDIRs* using RNA-seq

Gene expression level data of different tissues were downloaded from MDSi: Multi-omics Database for *S. italica* (http://foxtail-millet.biocloud.net/page/tools/expressionVisualization) (Yang et al., 2020; Li et al., 2023). The heatmap was generated by TBtools software (Chen et al., 2020).

### 4.8 RNA extraction and quantitative real-time PCR

The primer 5.0 software was used to design specific primers for *SiDIR* genes in this study (**Supplemental Data 7**). Total RNA was extracted using the KKFast Plant RNApure Kit (ZOMANBIO, ZP405K-2). The cDNA was synthesized by PrimerScript™ IV 1st strand cDNA Synthesis Mix (TaKaRa, Mountain View, CA, USA; 6215A). The quantitative real-time PCR (RT-qPCR) system program was performed according to the previous research (Duan et al., 2023). The gene expression was analyzed by the 2^−ΔΔCT^ method as used in a previous study (Gong et al., 2022).

### 4.9 Subcellular localization analysis

The CDS sequence of *SiDIR7*/*19*/*22* was cloned from the cDNA of *S. italica* by the primers of SiDIR7-F/R, SiDIR19-F/R, and SiDIR22-F/R, respectively. The vector of 35S-YFP was digested with *Bam*HI. The amplified products were then inserted into the linearized carrier of 35S-YFP by the kit of ClonExpress^®^ MultiS One Step Cloning (Vazyme, Nanjing, China; C113) and verified by DNA sequencing. These four vectors were transformed into *Agrobacterium tumefaciens* strain GV3101. *Agrobacterium* cultures harboring each construct were resuspended and mixed before being infiltrated into *Nicotiana benthamiana* leaves as described previously (Gong et al., 2022). After 48 h, the fluorescence signals were detected using a Leica TCS SP8 confocal microscope, and images were captured by LAS‐X software (Leica, Wetzlar, Germany).

## 5 Conclusions

In summary, 38 *SiDIR* gene family members were identified. We investigated the important role of *SiDIR* genes through the analysis of gene structure, evolutionary history, tertiary structure, *cis*-elements, stress responses, protein interaction, co-expression network, subcellular localization, and potential function. This study provides significant evidence and profound insights into the functional diversity of DIR proteins. Millet, a model for C_4_ photosynthesis, is one of the most traditional staple foods and the most economical and important source of energy for humans. This research may lay the foundation and pave a new way for improving the abiotic tolerance and agronomic traits of millet.

## Data availability statement

The original contributions presented in the study are included in the article/**Supplementary Material**, further inquiries can be directed to the corresponding authors.

## Author contributions

LG, BL, and TZ conceived the idea. LG and BX wrote the first draft. LG and BX corrected the paper to the present form. All authors contributed to the article and approved the submitted version.

## Supplementary material

Supplementary Figure 1 Predicted protein-protein interaction network for SiDIR based on their orthologs of AtDIR.

Supplementary Data Sheet 2 Multi sequence alignment within the conserved motifs of SiDIRs.

Supplementary Data Sheet 7 Primer sequences used in this study.
