# Supplementary figures and images for: Genome-wide identification and expression profiling analysis of DIR gene family in Setaria italica
Source: Front Plant Sci. 2023 Sep 20;14:1243806. doi: 10.3389/fpls.2023.1243806 (PMC10548141; doi:10.3389/fpls.2023.1243806)

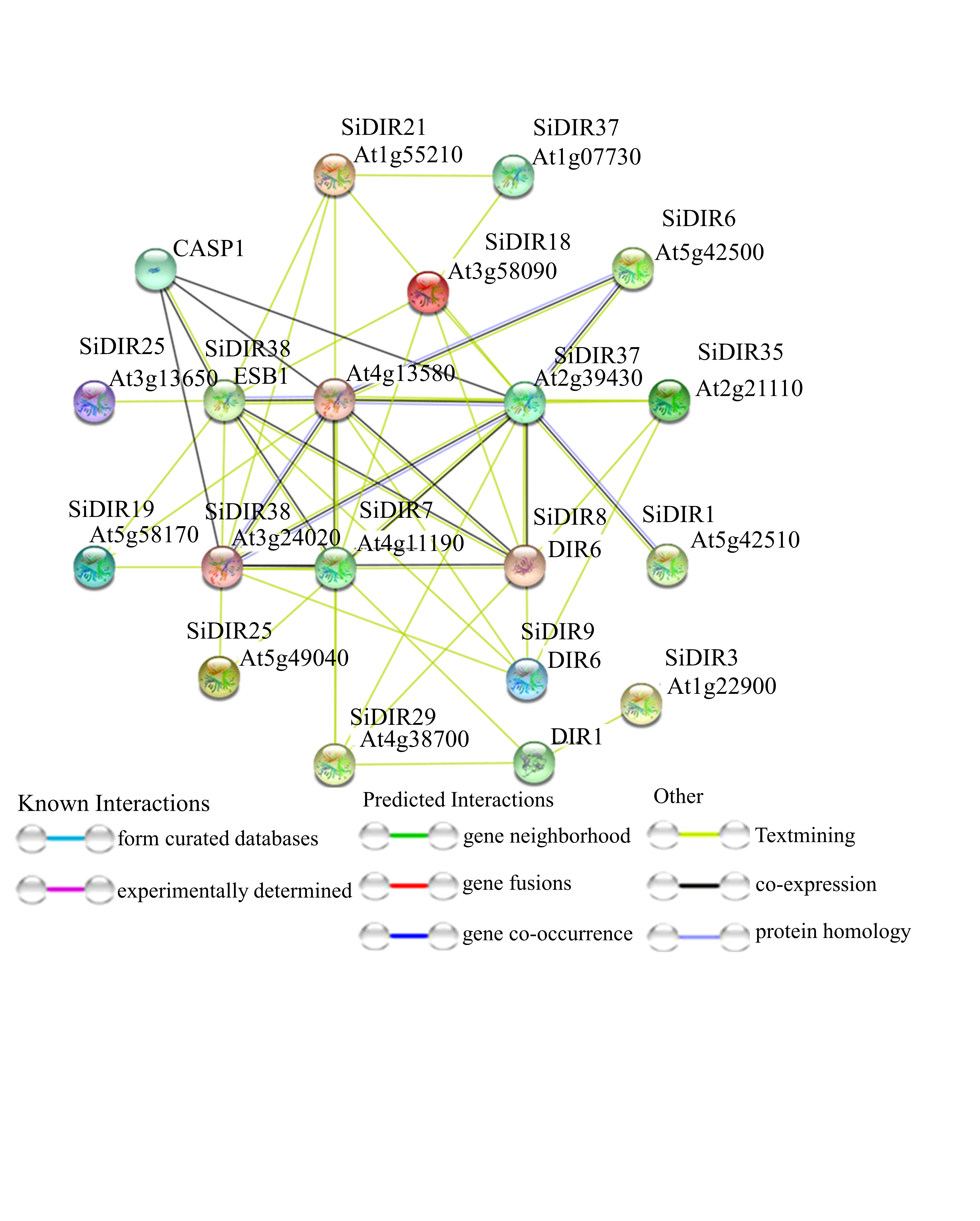

Supplement: Supplementary Figure 1 — Predicted protein-protein interaction network for SiDIR based on their orthologs of AtDIR. [file Image_1.tif]
