# Supplementary material for: Genome-wide identification and expression profiling analysis of DIR gene family in Setaria italica
Source: Front Plant Sci. 2023 Sep 20;14:1243806. doi: 10.3389/fpls.2023.1243806 (PMC10548141; doi:10.3389/fpls.2023.1243806)
Supplement: Supplementary Data Sheet 2 — Multi sequence alignment within the conserved motifs of SiDIRs. [file DataSheet_2.pdf]

```
SiDIR3   ............................................................
SiDIR34  ............................................................
SiDIR33  ............................................................
SiDIR1   MLLKEVVNSLHEVSFYHFPTAFEEPTRRPGENEGEWLCPDAEARIGLGLGKAESWNVLLI
SiDIR29  ............................................................
SiDIR30  ................................................MFKKDRIANICT
SiDIR20  ............................................................
SiDIR21  ............................................................
SiDIR19  ............................................................
SiDIR25  ............................................................
SiDIR26  ............................................................
SiDIR23  ............................................................
SiDIR22  ............................................................
SiDIR27  ............................................................
SiDIR35  ............................................................
SiDIR13  ............................................................
SiDIR14  ..............................................EASQRAAGASWREK
SiDIR15  ............................................................
SiDIR18  ............................................................
SiDIR24  ............................................................
SiDIR6   ............................................................
SiDIR37  .........................................................MAT
SiDIR38  ............................................................
SiDIR7   ............................................................
SiDIR8   ............................................................
SiDIR9   ............................................................
SiDIR2   ............................................................
SiDIR5   ............................................................
SiDIR10  ............................................................
SiDIR11  ............................................................
SiDIR4   ............................................................
SiDIR17  ............................................................
SiDIR32  ............................................................
SiDIR31  ............................................................
SiDIR12  ............................................................
SiDIR16  ............................................................
SiDIR36  ............................................................
SiDIR28  ............................................................
```

```
SiDIR3   ............................................................
SiDIR34  ............................................................
SiDIR33  ............................................................
SiDIR1   DRYDAFLFHLKWIVTSRNGPTTIVNSSSRLIARSTLQKPDHHLLAASNVASIHQGRAPMA
SiDIR29  ............................................................
SiDIR30  IRPHVRMKSLKRVETGYAGVVYKHGYATEQTHYQGRKQNLSLKAQASNPPSIMMPSLP.S
SiDIR20  ..........................................................MA
SiDIR21  ..........................................................MA
SiDIR19  ..........................................................MA
SiDIR25  ...........................................................M
SiDIR26  ...........................................................M
SiDIR23  ..........................................................MA
SiDIR22  ..........................................................MA
SiDIR27  ..........................................................MA
SiDIR35  ...........................................................M
SiDIR13  ...............................................TSISQTHHNGQLC
SiDIR14  EGAVGEIGGVRSGREERRSHVSLTAAHVRSAAAAAAGMGDSGGSVVSVDVERISFGGKLC
SiDIR15  .............................MKLLLLSPTIINPGYIPATSISQTHHNGQLC
SiDIR18  ..........................................................MA
SiDIR24  .........................................................MVP
SiDIR6   ........................................................MASS
SiDIR37  RNAVQAVRAALYLALALAVANCAFAGRVLDEQPQPAAPAEAPLPDDPLPAPTDPPTDPVV
SiDIR38  ............................................MLSFPSPAVPLTVLLI
SiDIR7   ................................................MQGLMPTCKLIT
SiDIR8   ................................................MQGHAPSSKFIV
SiDIR9   ................................................MQALKPSSSKLT
SiDIR2   ..........................................................MT
SiDIR5   ...........................................................M
SiDIR10  ............................................................
SiDIR11  ............................................................
SiDIR4   ............................................................
SiDIR17  ............................................................
SiDIR32  ............................................................
SiDIR31  ...........................................................M
SiDIR12  .........................................................MAA
SiDIR16  ...........................................MAATRAAKSLLLCLMVA
SiDIR36  ..................................................MLARIIFCVV
SiDIR28  ...........................................................M
```

```
          1       10        20                            30
SiDIR3    MACCKLFSIMLLAAVLAAGPAVA.....................AATSAHLHFYMHDVTG
SiDIR34   MACCKLFSIMLLAAVLAAGPAVA.....................AATSAHLHFYMHDVTG
SiDIR33   MASCKLYSVALLVVILAAGPATRPV...................SAASAHLHFYMHDVLT
SiDIR1    PSLAAAVLLFLLAPMSSPAAVLAAD...................GGTTTHLHLFMHDILT
SiDIR29   ..MASVMRHVQHLLAVAAIAAVATH...................GVTTTHLQFYMHDTVT
SiDIR30   VFLSAAFLLAAAAYIRSSGTVGSST...................ATSTTHLHFFMHDDYT
SiDIR20   AAVPLLLLPLLLATTAASAASSGG....................GEKQTHIKLYWHDVVS
SiDIR21   AAAATALLLLPILAAAPWAASAAS....................GEKSTHMKLYWHDVVS
SiDIR19   AAAAATLLLPLLLSAMITASVAAA....................GEQETLIKVYWHDVVS
SiDIR25   AGTSFAALSCLFAVALLAATASAA....................GEKETRLRVFWHDVVS
SiDIR26   AGTSFAALSCLFAVALLAATASAA....................GEKETRLRVFWHDVVS
SiDIR23   GSSSLAALSCVLAVALLAATASAE....................GEKETRLRVFWHDVGS
SiDIR22   MATTVLLLCAAVALAPTPSSAADD....................AAGFTTFKLYFHDIVG
SiDIR27   KKAVALLLFLVLVVAAEAHHPEAHDGG............RHKDAAAAPTHLHFYFHDTVS
SiDIR35   RSPSLLLLAILLGAAVSLQSAADTPEL............LG...AEKTTRLRFFLHDTLS
SiDIR13   KAAASARLLLLVAVIVLLLPSADAR....................RRPVHLRLYMHDIIG
SiDIR14   KAAASARLLLLVAVIVLLLPSADAR....................RRPVHLRLYMHDIIG
SiDIR15   KAAASARLLLLVAVIVLLLPSADAR....................RRPVHLRLYMHDIIG
SiDIR18   IWLLSLLVMALSSTTTVLASSGDPGPH............PFGDQGGSLTRIHLYVHETFK
SiDIR24   PKLQLPLLLSLLASVAVVHVAGDAG...................HDGLTHIHLYVHETYT
SiDIR6    PSLLVLVVALLAVFSPAVLASGREE......................RARIRVYVHERFS
SiDIR37   VAPAAGPAAAGAAGAATAGAAATGNAAGAASGGAAAANVGAAGAGAGDHPLTFFMHDILG
SiDIR38   ATLHVAAVHAQIPAAATTGAAVAATNP..QSGSAGGVGGAGAGAGGPDAPLELYMHDILG
SiDIR7    VIP.AILLLLGLMTGVAHGRRRLVSSH................DGEPCLNMTVYYHDILY
SiDIR8    ISSIAVMLLLGQLAGVAHGGGRQRN....................PPCKEMTVYYHNILY
SiDIR9    LIVASVVFLLGLAGVAHGGRRLISSHD................DNKPCKKMTVYYHDILY
SiDIR2    NPPYSKFGALRNPPQEKELFFH..............................LYLHHAHS
SiDIR5    AQPYFEIASAPCPLQRNEFYMH..............................LYLRQTGS
SiDIR10   .MANFQITQSPLPDENNEFNFSN.............................LYLFHTHS
SiDIR11   .MANFQITPSRVTVDNNEYNFSN.............................LYLLHAPS
SiDIR4    .......................MKKNELKISN.............................LYLHHAYR
SiDIR17   ......MASYTISPVQSELKMT..............................LYNKEVYA
SiDIR32   ......MASYTIDSVQSELKMT..............................LYIKEVYD
SiDIR31   AANPSYYQTSASVTSPVQHQEHLFH...........................LYAFQQYG
SiDIR12   APCPSLVLVLLLICSCAAADGELIH........................HLHFYFHEVDT
SiDIR16   FLHHLQFQFPPVAATTSLGKNESRDGG................SHGKTLSFTLYQQETIN
SiDIR36   VAAAVLAVVLLATVSPLPHGSGGGHRG.................ATGQRSFTVYVHPTVS
SiDIR28   ATLSNSLIFLCFLLLPAVLAFEGPHR....................ESYPCPYNCPPSKE

                                                         40        50
SiDIR3    ...............................................GPSPTAVRVVSGP
SiDIR34   ...............................................GPSPTAVRVVNGP
SiDIR33   ...............................................GPSPTAVQVLNGP
SiDIR1    ...............................................GSNPTAVQVIKGP
SiDIR29   P............................................SAGSPATAVRVVRGP
SiDIR30   ...............................................GPRPTAARIVSGR
SiDIR20   .............................................GPS..PTAVQVAHAA
SiDIR21   .............................................GPS..PTAVPVARAA
SiDIR19   .............................................GPD..PTVVKVARAA
SiDIR25   .............................................GGPNVSTVVQVAEGP
SiDIR26   .............................................GGPNVSTVVQVAEGP
SiDIR23   .............................................GGPNVSTVVLVAKGP
SiDIR22   .............................................GTS..PTAIRIAQAP
SiDIR27   .............................................GKS..PTAVRVLDPP
SiDIR35   .............................................GKD..PSAVLVARGE
SiDIR13   ...............................................GPEQTAIHLIRNA
SiDIR14   ...............................................GPEQTAIHLIRNA
SiDIR15   ...............................................GPEQTAIHLIRNA
SiDIR18   ...............................................GANSTAIRPVASP
SiDIR24   ...............................................GANATAAAVLQSP
SiDIR6    ...............................................GSNATVGSVAASP
SiDIR37   GSQPSGRIVTGVVASAAANGQLPFARPNTNIFPIQG.AVPLPQGATSLINGNNAPYVAGL
SiDIR38   GSSPTARPITGLLG.NIYNGQVPFARPIGFSAPRNGVAIPNANGQVPTVNGNNG..IPLD
SiDIR7    D..........................................GTN.TANATAAAATQP.
SiDIR8    D..........................................GTN.TANATAAFATQP.
SiDIR9    N..........................................GSNNTANATSAAVTKPS
SiDIR2    ...............................................GADQNQFEILASK
SiDIR5    ...............................................GSDRTQAEIVPPK
SiDIR10   ...............................................GPNRNQFSVTSED
SiDIR11   ...............................................GPRKNQAEVTRSD
SiDIR4    ...............................................EPSPTHLTLLSPK
SiDIR17   ...............................................GAGINGVTVINKE
SiDIR32   ...............................................GPDTNGVTIISRA
SiDIR31   ..................................................TNEHNIVPSK
SiDIR12   G...........................................TRHAQRHLHQRRQPAP
SiDIR16   ...............................................KTAYIVVDGVAGA
SiDIR36   .................................................AAVQAQQQGKE
SiDIR28   ...............................................THLRMYSHQFPAS
```

```
                                                        60        70
SiDIR3    .......................................RGYFGNTVVIDDKLTEGTSRS
SiDIR34   .......................................RGYFGNTVVIDDKLTEGTSQS
SiDIR33   .......................................RGHFGDTVVIDDVLTESASRS
SiDIR1    SAVP...................................GLAFGDTTVIDDALTEDPSSS
SiDIR29   TPAPGDP................................INRFGDLYVIDDPLTEGPDLA
SiDIR30   SLLPTLASSSDGDDNATATGSTLSRLL..........SSPRQFGDVVVLNNALTEGPGGD
SiDIR20   VTN..................................TSKTGFGAVVVIDDALTEGPDLK
SiDIR21   VTN..................................NSKTAFGAVVVIDDPLTQGPDLK
SiDIR19   TTN..................................ASKTLFGSVMVIDDPLTEGPDLG
SiDIR25   NSN..................................ASATGFGSVTVIDDPLTEGPNLT
SiDIR26   NSN..................................ASATGFGSVMVIDDPLTEGPNLT
SiDIR23   NSD..................................ASATRFGYVTVIDDPLTEGPNLT
SiDIR22   SSN..................................SSSTFFGAVVAIDDPLTSGPTRA
SiDIR27   SSS..................................SPMTMFGMVNVMDDPLTEGPEQE
SiDIR35   GRTPKP...............................EDPVPFSSLYATDDLLTEGPEPG
SiDIR13   GPPHKS...............................LKGAYFGDTMAIDDLVTDGPGID
SiDIR14   GPPHKS...............................LKGAYFGDTMAIDDLVTDGPGID
SiDIR15   GPPHKS...............................LKGAYFGDTMAIDDLVTDGPGID
SiDIR18   LGA....................................NSSFGSVGVLDDELRVGRDRA
SiDIR24   LGA....................................NSSFGSIGVVDDEIRVGPDRS
SiDIR6    LGD....................................NSTFGEVGVVDDVLRAGPDPS
SiDIR37   GGTSGGGVLVQNNGNPVNGGNKNIPFVNAGDLPSGVTLQNLLFGTTTVIDDELTEGHELG
SiDIR38   TGLSGAGFLQPGSGAAAAAAPAQVQLG.....PDGLSLG...FGTITVIDDVLTSGPDLG
SiDIR7    TLLSR.............................SVSINDTYFGEIVVFNDVVTAAPALA
SiDIR8    TLLSR.............................SASINDTYFGEIVVFDDPVTAGPALA
SiDIR9    AALSR.............................SNWSNGTFFGMLVVFDDLVTEEQALT
SiDIR2    RPN......................................ILGTTLVNDWLIYDK.RGQ
SiDIR5    EPN......................................SFGVTHVIDWPIAVV.PEP
SiDIR10   PAT......................................GLGQIAVNNWEIYDG.VGE
SiDIR11   ADT......................................GLGVIAVNNWEIYDG.LGP
SiDIR4    GQS......................................GFGATVSNNWAIHDGPDLS
SiDIR17   P.........................................MGTTWVFSWPVTDG.PGT
SiDIR32   K.........................................MGTTWVFSWLVTDG.PGP
SiDIR31   GLP.....................................NHFGCTNVTDWDIRDAPDNK
SiDIR12   ARVRN...............................TPSASTFGDVNVFDDELPEGPDPA
SiDIR16   GVS..................................QTTTPFGTIYVFRDYLTVRADRA
SiDIR36   VAR.....................................RGEASALVFHHRMTAGPEST
SiDIR28   GAN...................................PNEVAWPGWAADHWLLTWGPDP
```

```
                 80        90        100       110       120
SiDIR3    S.ATVGRAQGYYMVAS.VANLELLVSMNVVLTSG.PYAGSSLTVVGRDDVGTP.......
SiDIR34   S.ATVGRAQGYYMVAS.VANLELLVNMNVVLTSG.PYAGSSLTVLGRDDIGTP.......
SiDIR33   S.SAVGRAQGHYIWAS.TGNPELLVTMNVVLTSG.PYAGSSVTVVGRDDIGAP.......
SiDIR1    S.AAVGRVQGFYMMTSQSGGAVLTVCANLLLTAG.GYNGSTVAVMGRDDIAAD.......
SiDIR29   S.RAVGRAQGFYLMAS.RSIDQLLLSANMAFTAG.KYNGSSITLLGRDAIFDE.......
SiDIR30   S.ARVGTAQGFAVRVS.EGGIVSHLTMHMVLDAG.EHRGSSVTANGRIDMDAT.......
SiDIR20   SSKPLGRAQGTYIAAG.KDEVSLMMNMNFVFQAG.KYNGSTVAIMGRNAVFNA.......
SiDIR21   SSKPLGRAQGTYIAAG.KDELSLMMNMNFVFQAG.KYNGSTVAIMGKNAAFDA.......
SiDIR19   SSRLVGRAQGTYVSAG.KDVPELAMAMTFVFQGGSRYNGSSVAIMGRNEVGAD.......
SiDIR25   S.RLLGRAQGMYVSAG.KDSLSLMMAMTFVFVDG.AYNGSSLAILGPNQADRK.......
SiDIR26   S.RLLGRAQGMYVSAG.KDSLSLMMAMNFVFVDG.AYNGSSLAILGPNPAERK.......
SiDIR23   S.RLLGRAQGMYVSAG.KDSMSLLMAMNFVFVDG.AYNGSSLAILGPNAVERK.......
SiDIR22   AGTEVGRAQGTYTFAD.QKTFGLLMVMNFVFTAG.EYNGSSLSILGRNEVIND.......
SiDIR27   S.NPVGRAQGLYMGSD.QAKLGFLQAMNLVFTSG.DFNGSTLALLGRNCPLDA.......
SiDIR35   S.KVVGNAQGLYISSG.RGKLSLVLGMDFELTDG.PFNGSAFVIYSRNTVVTHPV.....
SiDIR13   S.GSVGRAQGTYMLSS.QHEEVLVAAVTVALTDG.PCSGSTFVIAGRVGIYDD.......
SiDIR14   S.GSVGRAQGTYMLSS.QHEEVLVAAVTVALTDG.PYSGSTFVIAGRVGIYDD.......
SiDIR15   S.GSVGRAQGTYMLSS.QHEEVLVAAVTR..........QHLLVAGRVGIYD........
SiDIR18   S.ELLGRFQGLVLGTGLEGGANYLTSVTFVFTAG.DYQGSTLSVVGPVLG.FKG......
SiDIR24   S.QLVGRYQAVFFGTSLQLGAGYLSSVTLVFTAG.EHAGSTLSVQGPILG.FTG......
SiDIR6    S.REVGRYQGLFAGADLED.ASYFSAITLVFTAG.EHRGSTVSLQGKYGFPVDG......
SiDIR37   A.GVIGRAQGFYVASS.QDGTSKTIVLTAMFEGPDAPHGDTLSFFGVHRMAAP.......
SiDIR38   A.QPLGRAQGVYVASS.ADGSSQMMAFTAMMEG..GEYGDTINFFGVYKVGTP.......
SiDIR7    S.APVARAEGFYFYDR.KEALSAWFAFSLVFNS..TAHRGTLNLMGADLMAEK.......
SiDIR8    S.EPVARAQGFFFYDK.KESPNAWFAFSLVFNS..TAHRGTLNLMGADLMAEK.......
SiDIR9    S.EPVARAQGFYLYDK.KEAYNVWIAFTLVFDS..KAYKGTLNLMGADLMAEE.......
SiDIR2    GAKVVARAQGLHIQSGMKA.KNWRSSFSIVFEDA.NARFKGSSLEVMGPHIRFG......
SiDIR5    AATIVARAQGLLMQAGLIN.PRTYTSFNIVFEDD.RRKSNN...................
SiDIR10   STTIVARAQGMHIHA.....GNWTNVFSIVFEDK.RFRGSTLQVMGISVDENG.......
SiDIR11   GAAIVARAQGLHIHA.....GNWSNVFSIVFETP.RFSGSTLEVMGTSVDIG........
SiDIR4    KDAIVARSQGLHMQS.....GNWHNSFTIAFEID.GLKDSTLQVMGLGVDKGTD......
SiDIR17   NANIVGHLQGTGVQVANTPNYVWHYSLGLVFGDK.RFNGSTLQISGTSQING........
SiDIR32   DANIVGHMQGTSVQVANTP.DVYQYSLGLVFGDK.RFNGSTLQISGTSQING........
SiDIR31   A.TVVARLQGVGIAAR.KSTESWYGSFIVVFTDQ.RFKGSTLSVQGPLGPATLGD.....
SiDIR12   S.RLIGRARGLAAHAS...LDESRGLSPVDFDR...........................
SiDIR16   S.PVAGVAEGSSITTTLDGLQSLSLAKITVDHRG..HRGSVSVLGGTYNTRPS.......
SiDIR36   S.RTVGAASGFVLLLAGERGLAAASVFDTVHLSFDGAAGLSGSLCVEACSRRPEKRGHDA
SiDIR28   NQNIAGRARGFHLLTG.ETGKDWYISHIYVFQDDSRFAGSTIQVLGMLNG..........
```

```
                 130       140       150       160
SiDIR3     .VRELSVVGGTGQFR.MARGYVLWKTVTP......EILDLEIFVNP..............
SiDIR34    .LRELSVVGGTGQFR.MARGYVLWKTITP......EILDLEIFVNP..............
SiDIR33    .VRELSVVGGTGRFR.MARGYVLWKTVSLDHPN..AVLELDVFVDA..............
SiDIR1     .VRELAVVGGTGRFR.MATGYVLWKTNSMNGPD..ATVELDVYVTTGGGATIDSSTPVGG
SiDIR29    .IRELPVVGGTGGFH.GAAGYGLIRTHSLNASN..NNAVLVIDMYLML............
SiDIR30    .VRESVVIGGTGKFR.FARGYMLTRNYDYDLARG.GIVEIDVYVQH..............
SiDIR20    .VREMAVVGGTGVFR.MARGYAQARTHTFDLKTGDATVEYNIYIKH..............
SiDIR21    .VREMAIVGGTGVFR.MARGYAQARTHTLDLKTGDATVEYNLFIKH..............
SiDIR19    .VREMAVVGGTGVFR.WARGYAQARTHAFNLSTGDATVEYSLFVRH..............
SiDIR25    .VREMAVVGGTGVFR.FAHGYCEARMRWFDARTGDATVEYSIHVRHD.............
SiDIR26    .VREMAVVGGTGMFR.FAHGYCEARTRWFDARTGDATVEYSIHVRHD.............
SiDIR23    .VREMAVVGGTGVFR.FANGYCEARTQWIDTRTSDATVEYNIHVRHD.............
SiDIR22    .VREMSIVGGSGKFR.MARGYVQAHTIDSGATSGETVVQYTVNVKA..............
SiDIR27    .VRELPVVGGTGAFR.FARGYAQLRTHWLDFRTGDATVEYDVYVMH..............
SiDIR35    .GRELAIVGGRGKFR.MARGYALLRTHVLDNNNGDAIIEYNVTLHHH.............
SiDIR13    .KSELAIVGGTGQLR.RAAGYVLWR.MAKVVSEVYIVVELIVHMSVPANAAAPSNGSSLA
SiDIR14    .KSELAIVGGTGQLR.RAAGYVLWR.MAKVVSEVYIVVELIVHMSVPANAAAPSNGSSLA
SiDIR15    .KSELPIVGGTGQLR.RAAGYVLWR.MAKVVSEVYIVVELIVHMSVPANAAAPSNGSSLA
SiDIR18    .AIERPVVGGTGQVQDGQRGYSMLKLLGNPTPETVLFEVDLFVLVHRGKY..........
SiDIR24    .TIERAVVGGTGKFR.LARGYMLFKMISKPTPETDVNEVDLFVLMHHGKY..........
SiDIR6     .ALERVVVGGTGGFR.MARGFSMLKVVSN.TPEADVFQLDLVVFTPRRR...........
SiDIR37    .ESHIAIIGGTGKYE.NAKGFAAIQTLHPGDEHTTDGVETLLQFNIHLI...........
SiDIR38    .LCRLSITGGTGKFK.GACGFAEVRPLIASGQHVTDGAETLLRITVHLA...........
SiDIR7     .TRDISVVGGTGDFF.MARGVATLRTDTFQGLYYFRLQMDIKLYECYV............
SiDIR8     .TRDISVVGGTGDFF.MARGVATLRTDSYEGLYYFRLQMDIKLYDCYDV...........
SiDIR9     .TRDLSVVGGTGDFF.MSRGVATLSTNATEGYFYFRLKMDIKLYECYVA...........
SiDIR2     ...QWAIVGGTGEFT.LARGIIHKTEVER..LRDKNIIELKIHAFYTPMRA.........
SiDIR5     ...QWAISSGTGGFA.LAHGIIRQKVMPLDGRRDTNIKELHIHAFYTPMNN.........
SiDIR10    ...EFAIVGGTGQFA.MATGVISKKLHQR..SNDGTIVQLTIHGFSPVLKGWSPPP....
SiDIR11    ...EFAIVGGTKQFA.MARGVIYKKYLPEQSTSDGGIIQLTIRGFFPVLKPQPSPPPPPP
SiDIR4     ...QWSIVGGTGQFT.FAQGFINKKLHKV..VDTGNIIELDIYAIFQTKYT.........
SiDIR17    ...EWSIVGGTGELS.MAKGTVTRTEITN..TGNTRISELKIHAFYTPMNR.........
SiDIR32    ...EWSIVGGTGELA.MAKGTIKRTEIRY..IGNTRISELKIHAFYTPMNS.........
SiDIR31    .EGDWAVVGGTGEFV.YAQGVCSYKRIQAISGVLINELRIRVMCLTIPMPKPVQKIG...
SiDIR12    ........LLRLPAALR.FARGYMHDQQALSATDTAIVVVFDIHVPARAFDPN........
SiDIR16    ...DYPVVGGTGDFA.YALGYVRSSPVDLRGRTVTYKMELHLYWPPYAHYAPVPHKPV..
SiDIR36    EEEVLQVVGGTGAFA.FARGHAVLRRQRPGHGVTAAALCLDISVFSAAS...........
SiDIR28    ...EWSIIGGTGAFY.NARGYIKYKEVPSTIISNITDIVRELDVHIFTRETSTVANGGPV

SiDIR3     ............................................................
SiDIR34    ............................................................
SiDIR33    ............................................................
SiDIR1     SSSAAAVRVGGWVSAVFVAVVVAVVGSYV...............................
SiDIR29    ............................................................
SiDIR30    ............................................................
SiDIR20    ............................................................
SiDIR21    ............................................................
SiDIR19    ............................................................
SiDIR25    ............................................................
SiDIR26    ............................................................
SiDIR23    ............................................................
SiDIR22    ............................................................
SiDIR27    ............................................................
SiDIR35    ............................................................
SiDIR13    IE..........................................................
SiDIR14    IE..........................................................
SiDIR15    IE..........................................................
SiDIR18    ............................................................
SiDIR24    ............................................................
SiDIR6     ............................................................
SiDIR37    ............................................................
SiDIR38    ............................................................
SiDIR7     ............................................................
SiDIR8     ............................................................
SiDIR9     ............................................................
SiDIR2     ............................................................
SiDIR5     ............................................................
SiDIR10    ...SQVKKIEPLGGGGGVVQDITEAPG..RLESITVQSGVVIDAIAFSYVDQAGQQRTAG
SiDIR11    VNRTRVLKIGPCGGNGGVVWDIPGTPSPTRLESITISYGGVIDGIEFSYINQSGQRCTTG
SiDIR4     ...HTYTRDGPKGGDAGQAREPKYEPH..RLETIKIDHGDLIYSIEYSHIDQYGTKHTEG
SiDIR17    ............................................................
SiDIR32    ............................................................
SiDIR31    ........PWGGNGGTPYEIQGAEQPQRLESVTIYANNNFIQTIAFSYTDQSSQKRAVGP
SiDIR12    ............................................................
SiDIR16    ............................................................
SiDIR36    ............................................................
SiDIR28    PI..........................................................
```

```
SiDIR3   ............................................................
SiDIR34  ............................................................
SiDIR33  ............................................................
SiDIR1   ............................................................
SiDIR29  ............................................................
SiDIR30  ............................................................
SiDIR20  ............................................................
SiDIR21  ............................................................
SiDIR19  ............................................................
SiDIR25  ............................................................
SiDIR26  ............................................................
SiDIR23  ............................................................
SiDIR22  ............................................................
SiDIR27  ............................................................
SiDIR35  ............................................................
SiDIR13  ............................................................
SiDIR14  ............................................................
SiDIR15  ............................................................
SiDIR18  ............................................................
SiDIR24  ............................................................
SiDIR6   ............................................................
SiDIR37  ............................................................
SiDIR38  ............................................................
SiDIR7   ............................................................
SiDIR8   ............................................................
SiDIR9   ............................................................
SiDIR2   .................EAVSAGQWTLGP...............................
SiDIR5   .................SVVPGATDGKS................................
SiDIR10  PWGG.SGRNSETIQLGPSEFVKGISGTVGL.YRSCK.VIASLTFVTNVRTCGPYG..LGD
SiDIR11  RWCGKGGTRTQLINLGPSEFVKEVSGTIGA.YRHYNNIIRTLAIVTNVRTYGPFGNQLNG
SiDIR4   ..RGTEGSETGIIELGPTEFVHEVSGTLGK.CNNIYTVLSSLTIVTNLRTLGPYG.KETS
SiDIR17  .................TNVSG.TTGCKF.EKA...........................
SiDIR32  .................TTVSSNNTGCKF.QKA...........................
SiDIR31  WGGDAGKSKQPPIQFGPSETVKEIYGTTGNNYDGVHTVVTSLTIVTNVNTYGPYGKQAAG
SiDIR12  ............................................................
SiDIR16  ............................................................
SiDIR36  ............................................................
SiDIR28  ............................................................
```

```
SiDIR3   ....................................
SiDIR34  ....................................
SiDIR33  ....................................
SiDIR1   ....................................
SiDIR29  ....................................
SiDIR30  ....................................
SiDIR20  ....................................
SiDIR21  ....................................
SiDIR19  ....................................
SiDIR25  ....................................
SiDIR26  ....................................
SiDIR23  ....................................
SiDIR22  ....................................
SiDIR27  ....................................
SiDIR35  ....................................
SiDIR13  ....................................
SiDIR14  ....................................
SiDIR15  ....................................
SiDIR18  ....................................
SiDIR24  ....................................
SiDIR6   ....................................
SiDIR37  ....................................
SiDIR38  ....................................
SiDIR7   ....................................
SiDIR8   ....................................
SiDIR9   ....................................
SiDIR2   ....................................
SiDIR5   ....................................
SiDIR10  GTPFTVPVEDNHSVVGFFVRSKTYLDAIGVYVQPQ.
SiDIR11  TAPFSIPVQNNSSIVGFFARGQQFLDAIGVYVQETQ
SiDIR4   ESPFSLPEKKGGSVVGFFASTGVAVGALGVIVRQ..
SiDIR17  ....................................
SiDIR32  ....................................
SiDIR31  NTPFRVAAPNNHSIVGFYGRVGDVVDQIGAYVSPN.
SiDIR12  ....................................
SiDIR16  ....................................
SiDIR36  ....................................
SiDIR28  ....................................
```
